# Development and validation of the prediction score for augmented renal clearance in critically Ill Japanese adults

**DOI:** 10.1186/s40780-024-00394-2

**Published:** 2024-11-06

**Authors:** Ryusei Mikami, Shungo Imai, Mineji Hayakawa, Hitoshi Kashiwagi, Yuki Sato, Shunsuke Nashimoto, Mitsuru Sugawara, Yoh Takekuma

**Affiliations:** 1 Graduate School of Life Science, Hokkaido University, Sapporo, Japan; 2 Department of Pharmacy, Hokkaido University Hospital, Sapporo, Japan; 3 Faculty of Pharmacy, Keio University, Tokyo, Japan; 4 Department of Emergency Medicine, Hokkaido University Hospital, Sapporo, Japan; 5 Faculty of Pharmaceutical Sciences, Hokkaido University, Sapporo, Japan

**Keywords:** Augmented renal clearance, Prediction score, Intensive care unit, Japan

## Abstract

**Background:**

Augmented renal clearance (ARC) decreases the therapeutic concentration of drugs excreted by the kidneys in critically ill patients. Several ARC prediction models have been developed and validated; however, their usefulness in Japan has not been comprehensively investigated. Thus, we developed a unique ARC prediction model for a Japanese mixed intensive care unit (ICU) population and compared it with existing models.

**Methods:**

This retrospective study enrolled a mixed ICU population in Japan from January 2019 and June 2022. The primary outcome was the development and validation of a model to predict ARC onset based on baseline information at ICU admission. Patients admitted until May 2021 were included in the training set, and external validation was performed on patients admitted thereafter. A multivariate logistic regression model was used to develop an integer-based predictive scoring system for ARC. The new model (the JPNARC score) was externally validated along with the ARC and Augmented Renal Clearance in Trauma Intensive Care (ARCTIC) scores.

**Results:**

A total of 2,592 critically ill patients were enrolled initially, with 651 patients finally included after excluding 1,941 patients. The training and validation datasets comprised 456 and 195 patients, respectively. Multivariate analysis was performed to develop the JPNARC score, which incorporated age, sex, serum creatinine, and diagnosis upon ICU admission (trauma or central nervous system disease). The JPNARC score had a larger area under the receiver operating characteristic curve than the ARC and ARCTIC scores in the validation dataset (0.832, 0.633, and 0.740, respectively).

**Conclusions:**

An integer-based scoring system was developed to predict ARC onset in a critically ill Japanese population and showed high predictive performance. New models designed to predict the often-unrecognized ARC phenomenon may aid in the decision-making process for upward drug dosage modifications, especially in resource- and labor-limited settings.

## Background

Augmented renal clearance (ARC) decreases the therapeutic concentration of drugs excreted by the kidneys in critical care settings [1–4]. This phenomenon is common in critically ill patients, and guidelines on sepsis and therapeutic drug monitoring increasingly recognize the importance of ARC [5, 6]. However, there remains a substantial unmet need to explore ARC in highly heterogeneous critical care environments.

ARC is most commonly defined as measured creatinine clearance (CrCl) > 130 mL/min/1.73 m^2^ [7–9]. ARC screening is essential because renal hyperfiltration increases the drug dose required to reach the therapeutic range. Although measured CrCl is useful for diagnosing ARC [8, 10, 11], the need for urine collection limits its frequent use in patients without renal dysfunction. Given the high prevalence of ARC (39%; 95% confidence interval [CI], 35–43%) in critically ill patients [10], an efficient method of identifying patients with ARC is needed.

Several predictive ARC scoring models have recently been developed and validated [12–15]. However, each of these models targets a specific population, such as patients with sepsis and/or trauma, and their applicability to broader critical care populations is yet to be adequately evaluated. Moreover, these studies were conducted in Western populations and did not consider racial differences. In particular, racial variations have been documented in some creatinine-based renal function estimation equations [16, 17], primarily because muscle mass, the source of serum creatinine, varies between racial groups. However, serum creatinine remains the standard endogenous biomarker for ARC prediction, given that ARC is defined based on CrCl. It is unclear whether these scores are useful in Japan as the serum creatinine thresholds predicting ARC may differ among Asians with lower muscle mass. Currently, there is a paucity of literature on ARC in the context of critical care in Japan. In this study, we aimed to develop and validate a predictive scoring system for ARC in a critically ill Japanese population.

## Methods

### Study design and setting

This retrospective study was conducted at Hokkaido University Hospital, a tertiary care hospital in Japan. It included critically ill adults admitted to the intensive care unit (ICU) between January 2019 and June 2022. This cohort was selected from a previous study [18], in which urinary CrCl was measured at least once in almost all initially screened patients. Patients younger than 18 years of age, those who received renal replacement therapy, those without a urine catheter, those discharged from the ICU within 72 h, and in-hospital emergency patients were excluded. Patients admitted until May 2021 were included in the training set, and external validation was performed on patients admitted thereafter.

### Study endpoints

The primary endpoint was the development and validation of a predictive scoring system for ARC (the JPNARC score) in a critically ill Japanese population. The model performance was validated using an external set, along with the ARC and Augmented Renal Clearance in Trauma Intensive Care (ARCTIC) scores.

### Definition of ARC and methods of evaluation of renal function

Based on previous studies, the cutoff for ARC was defined as CrCl > 130 mL/min/1.73 m^2^. The measured CrCl was calculated over a 6–24 h period. All equations for renal function evaluation are expressed per body surface area and calculated using the Du Bois formula [19].

### Model development and validation

Multivariate logistic models were used to identify factors associated with ARC onset based on baseline ICU admission information. Covariates were selected based on the ARC consensus from current literature and data availability [7, 8, 20, 21]. The covariates included age, sex (male), serum creatinine, diagnosis at ICU admission (trauma, central nervous system [CNS] disease [traumatic brain injury, intracerebral hemorrhage, subarachnoid hemorrhage, cerebral arteriovenous malformation, hydrocephalus, and status epilepticus]), sepsis, cardiovascular disease, and medical history (chronic kidney disease, hypertension, diabetes, myocardial infarction, heart failure, chronic obstructive pulmonary disease, cirrhosis, and liver failure). Continuous variables, such as age and serum creatinine levels, were categorized at thresholds where ARC was more common, using decision tree analysis to simplify the analysis [22].

The model performance was evaluated in terms of discrimination and calibration using a validation dataset. Predictive performance was compared with the ARC and ARCTIC scores. The ARC score consists of age ≤ 50 years (6 points), trauma (3 points), and a sequential organ failure assessment (SOFA) score ≤ 4 (1 point), with ≥ 7 points indicating a high-risk group [12]. The ARCTIC score uses age < 56 years (4 points), age 56–75 years (3 points), male sex (2 points), and serum creatinine < 0.7 mg/dL (3 points), with a total of ≥ 6 points applied as the cutoff for high risk [14, 15]. The discriminative ability of these models was evaluated using the area under the receiver operating characteristic (AUROC) curve. Additionally, the sensitivity, specificity, positive predictive value (PPV), and negative predictive value (NPV) were compared using the optimal cutoff for each score. The optimal cutoff for the JPNARC score was derived from the ROC curve of the development cohort using the Youden index [23], whereas the ARC and ARCTIC score cutoffs were based on thresholds established in previous studies [12–15]. Calibration performance was evaluated using a calibration curve. The goodness of fit was assessed using the Hosmer–Lemeshow test [24].

### Other statistical analysis

Continuous data are presented as median values (interquartile range), and categorical data as counts (%). The Mann–Whitney U test was used for continuous data analysis. The chi-square test or Fisher’s exact test was used for categorical data analysis. A *p*-value of < 0.05 was deemed statistically significant. All statistical analyses were performed using JMP version 17.0 software (SAS Institute Inc., Cary, NC, USA) and EZR (Saitama Medical Center, Jichi Medical University, Saitama, Japan), a graphical user interface for R (The R Foundation for Statistical Computing, Vienna, Austria) [25].

## Results

**Fig. 1:**
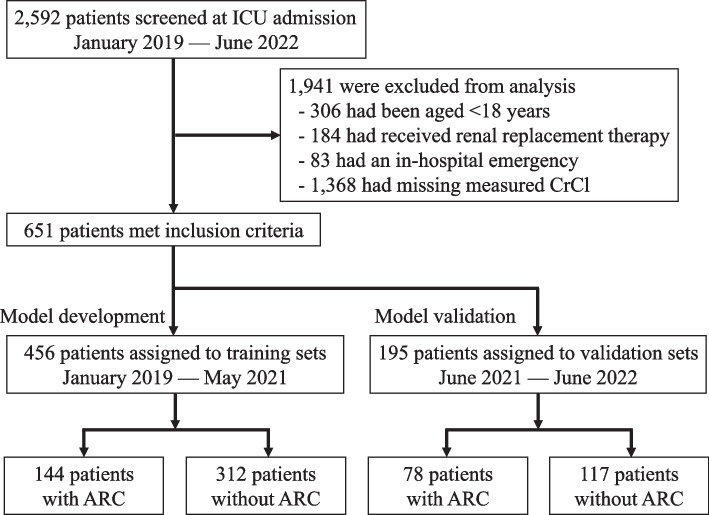
Selection flow of patients for analysis. ICU, intensive care unit; CrCl, creatinine clearance; ARC, augmented renal clearance

Of the 2,592 patients admitted to the ICU during the study period, 1,941 patients (306 patients aged < 18 years, 184 patients who received renal replacement therapy, 83 patients with in-hospital emergencies, and 1,368 patients with missing CrCl measurements [including patients without a urine catheter and those discharged early from the ICU]) were excluded, resulting in 651 patients who met the inclusion criteria (Fig. 1). A total of 456 patients admitted until May 2021 were used to develop the predictive model, and 195 patients admitted thereafter were used for external validation. The baseline characteristics and laboratory data of each population were typical of a mixed ICU cohort [26–28], without any sufficiently large differences that could impact the analysis (Table 1). The prevalence of ARC in the training and validation sets was 32% and 40%, respectively. The median (interquartile range) of measured CrCl versus estimated CrCl (Cockcroft-Gault equation [29]) for patients with ARC in the training set and validation set were 152 (140–177) mL/min/1.73 m^2^ versus 104 (86–137) mL/min/1.73 m^2^ and 150 (136–172) mL/min/1.73 m^2^ versus 99 (84–120) mL/min/1.73 m^2^, respectively.

**Table 1:** Clinical characteristics

	Total (*n* = 651)	Training set (*n* = 456)	Validation set (*n* = 195)	*p*-value
Age, years^a^	71 (57–80)	71 (58–80)	70 (55–79)	0.337
Male sex^b^	383 (59%)	266 (58%)	117 (60%)	0.692
Weight, kg^a^	57 (48–67)	57 (47–66)	59 (49–69)	0.081
Body surface area, m^2a^	1.59 (1.44–1.73)	1.58 (1.42–1.72)	1.62 (1.48–1.76)	0.066
SCr, mg/dL^a^	1.05 (0.78–1.49)	1.05 (0.76–1.49)	1.06 (0.83–1.48)	0.410
Admission diagnosis^b^				
Trauma	90 (14%)	65 (14%)	25 (13%)	0.627
CNS^*c*^	88 (14%)	60 (13%)	28 (14%)	0.681
Sepsis	68 (10%)	35 (8%)	33 (17%)	< 0.01
Cardiovascular	244 (38%)	182 (40%)	62 (32%)	0.050
Digestive	36 (6%)	23 (5%)	13 (7%)	0.407
Infection (without sepsis)	71 (11%)	48 (11%)	23 (12%)	0.634
Other	89 (14%)	68 (15%)	21 (11%)	0.159
SOFA score^a^	5 (3–8)	5 (3–8)	4 (3–8)	0.130
ICU-free days^a^	21 (11–24)	21 (11–24)	21 (12–24)	0.329
Mortality^b^	90 (14%)	57 (13%)	33 (17%)	0.134

*SCr* serum creatinine, *CNS* central nervous system, *SOFA* sequential organ failure assessment, *ICU* intensive care unit

^a^Median (interquartile range)

^b^Number (%)

^c^CNS refers to hospitalization for any of the following reasons: traumatic brain injury, intracerebral hemorrhage, subarachnoid hemorrhage, cerebral arteriovenous malformation, hydrocephalus, or status epilepticus

**Fig. 2:**
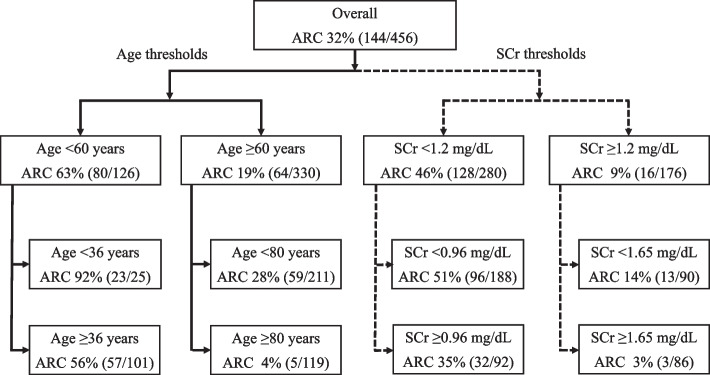
Decision tree analysis for setting thresholds of ARC-related factors. ARC, augmented renal clearance; SCr, serum creatinine

**Fig. 3:**
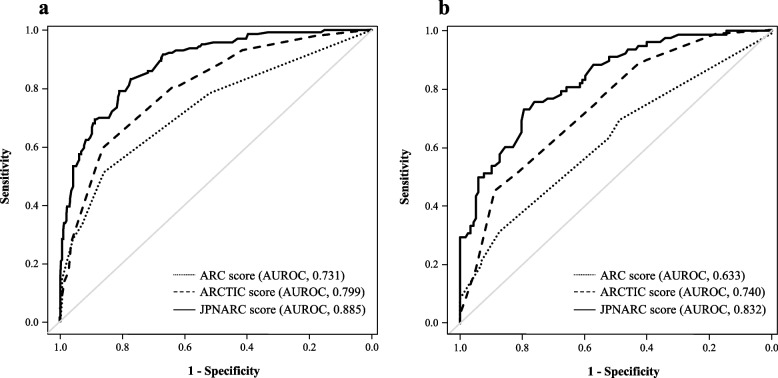
AUROC for ARC prediction scores of the (**a**) training and (**b**) validation sets. ARC, augmented renal clearance; ARCTIC, Augmented Renal Clearance in Trauma Intensive Care; AUROC, area under the receiver operating characteristic curve

A predefined multivariate logistic regression model was used to identify risk factors for ARC development in the training set (Table 2). At this time, the continuous variable covariates age and serum creatinine were classified by decision tree analysis at thresholds where ARC was more common (Fig. 2). Standardized regression (beta) coefficients from the multivariate analysis led to the development of the JPNARC score, which included age, sex, serum creatinine, and diagnosis at ICU admission (trauma and CNS disease) as risk factors (see Tables 2 and 3 for the score points and interpretations assigned to each factor). The AUROC (95% CI) of the JPNARC, ARC, and ARCTIC scores for the training set were 0.885 (0.853–0.916), 0.731 (0.681–0.780), and 0.799 (0.756–0.842), respectively (Fig. 3a). The Hosmer–Lemeshow test confirmed the goodness of fit of the model (*p* = 0.660). The optimal cutoff value for the ROC curve of the JPNARC score was 9.

**Table 2:** Identifying predictors of ARC by multivariate analysis

	Multivariate analysis			Score points
	OR (95% CI)	Beta	*p*-value	
Age				
﻿ < 36 years	96.3 (16.2–570)	4.6	< 0.01	7
36–59 years	17.6 (6.02–51.2)	2.9	< 0.01	5
60–79 years	8.35 (3.07–22.7)	2.1	< 0.01	3
≥ 80 years	Reference	–	–	–
Male sex	1.87 (1.04–3.64)	0.63	< 0.05	1
SCr				
< 0.96 mg/dL	21.2 (6.00–74.9)	3.1	< 0.01	5
0.96–1.19 mg/dL	9.82 (2.67–36.1)	2.3	< 0.01	4
1.20–1.64 mg/dL	3.69 (0.94–14.5)	1.3	0.06	–
≥ 1.65 mg/dL	Reference	–	–	–
Admission diagnosis				
Trauma	2.49 (1.12–5.55)	0.9	< 0.05	1
CNS^a^	3.18 (1.43–7.07)	1.2	< 0.01	2
Sepsis	1.20 (0.40–3.58)	0.2	0.74	–
Cardiovascular	0.77 (0.41–1.47)	-0.3	0.43	–
Medical history^b^	0.58 (0.33–1.01)	-0.6	0.06	–

*ARC* augmented renal clearance, *SCr* serum creatinine, *CNS* central nervous system, *OR* odds ratio, *95% CI* 95% confidence interval

^a^CNS refers to hospitalization for any of the following reasons: traumatic brain injury, intracerebral hemorrhage, subarachnoid hemorrhage, cerebral arteriovenous malformation, hydrocephalus, or status epilepticus

^b^Medical history of chronic kidney disease, hypertension, diabetes, myocardial infarction, heart failure, chronic obstructive pulmonary disease, cirrhosis, and liver failure

**Table 3:** Overview of ARC predictive scores

ARC score	Setting: Sepsis and trauma ICUs
	Criteria (score points): Age ≤ 50 years (6) Trauma (3) SOFA score ≤ 4 (1)
	Interpretation: ≥ 7 points: ARC high-risk
ARCTIC score	Setting: Trauma ICU
	Criteria (score points): Age < 56 years (4), 56–75 years (3) Male sex (2) SCr < 0.7 mg/dL (3)
	Interpretation: ≥ 6 points: ARC high-risk
JPNARC score	Setting: Mixed ICU
	Criteria (score points): Age < 36 years (7), 36–59 years (5), 60–79 years (3) Male sex (1) SCr < 0.96 mg/dL (5), 0.96–1.19 mg/dL (4) Trauma (1) CNS (2)
	Interpretation: ≥ 9 points: ARC high-risk

*ARC* augmented renal clearance, *ARCTIC* Augmented Renal Clearance in Trauma Intensive Care, *ICU* intensive care, *SOFA* sequential organ failure assessment, *SCr* serum creatinine, *CNS* central nervous system

**Fig. 4:**
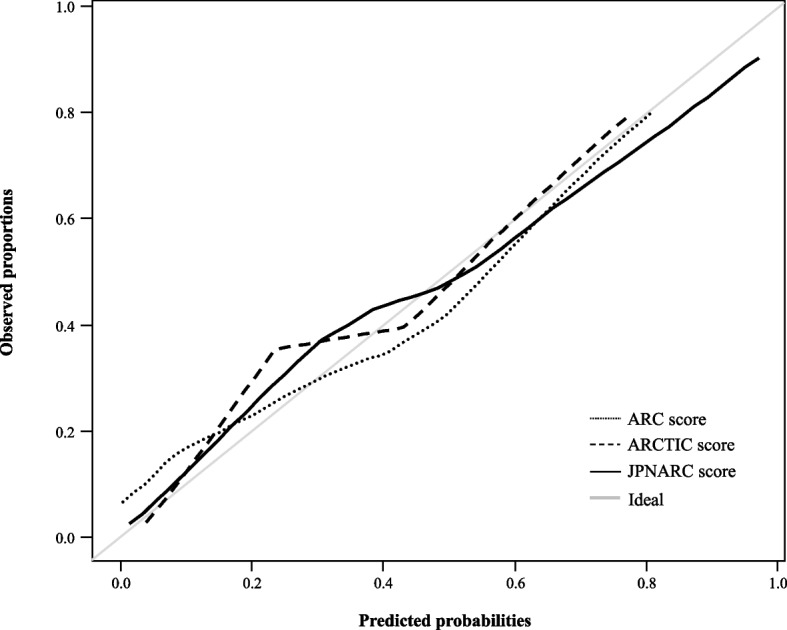
Calibration curves for ARC prediction scores of the validation set. ARC, augmented renal clearance; ARCTIC, Augmented Renal Clearance in Trauma Intensive Care

**Fig. 5:**
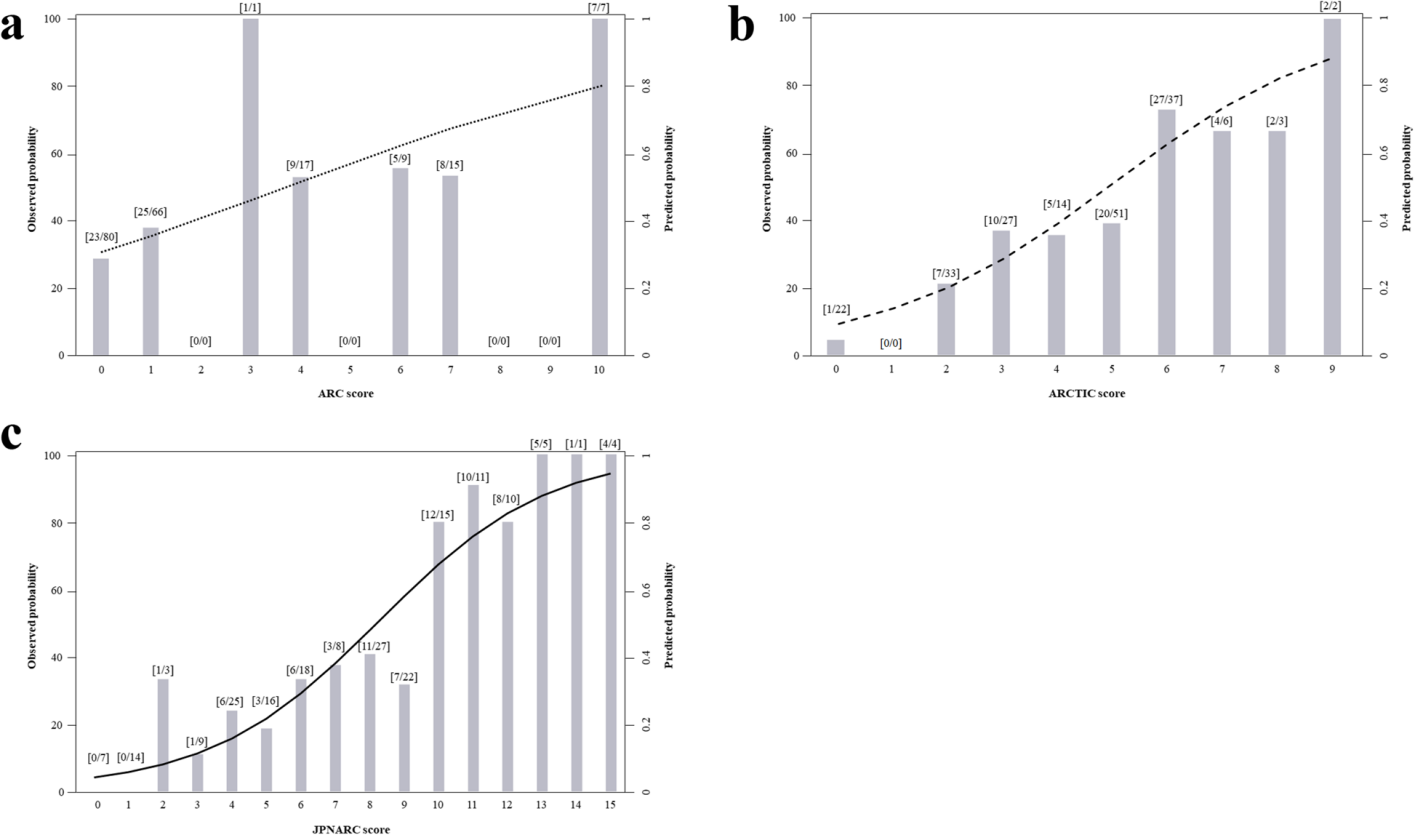
Relationship between predicted and observed probabilities from (**a**) ARC, (**b**) ARCTIC and (**c**) JPNARC scores of the validation set. ARC, augmented renal clearance; ARCTIC, Augmented Renal Clearance in Trauma Intensive Care

The performance of the JPNARC score was externally validated and compared with that of the ARC and ARCTIC scores. The AUROC (95% CI) of the JPNARC, ARC, and ARCTIC scores were 0.832 (0.775–0.888), 0.633 (0.556–0.710), and 0.740 (0.671–0.808), respectively (Fig. 3b). The sensitivity, specificity, PPV, NPV, calibration curve and predicted/observed probabilities for the JPNARC, ARC and ARCTIC scores are shown in Table 4, Figs. 4 and 5, respectively. JPNARC scores tended to overestimate the ARC occurrence in the ranges with high predictive probability, while ARC scores diverged between predicted and observed probability in a wide range and ARCTIC scores tended to underestimate it in the ranges with high predictive probability.

**Table 4:** Diagnostic performance of the ARC predictive scores

	Sensitivity	Specificity	PPV	NPV
ARC score ≥ 7 points	19%	94%	68%	64%
ARCTIC score ≥ 6 points	45%	89%	73%	71%
JPNARC score ≥ 9 points	60%	82%	69%	76%

*ARC* augmented renal clearance, *ARCTIC* Augmented Renal Clearance in Trauma Intensive Care, *PPV* positive predictive value, *NPV* negative predictive value

## Discussion

Herein, we developed a scoring system to predict ARC onset in critically ill adults in Japan (the JPNARC score) and validated its superior predictive performance. This new prediction score, comprising five risk factors—age, sex, serum creatinine, and diagnosis at ICU admission (trauma and CNS disease)—outperformed the existing scores in terms of availability and predictive accuracy. Upon comparing the predictive performance using optimal cutoff values for each score, we found that the JPNARC score was more sensitive than the other scores. This difference may be attributed to variations in the clinical settings and detailed thresholding of the score factors. The populations used to develop the ARC and ARCTIC scores consisted exclusively of patients with sepsis and/or trauma, with median ages of 42 and 48 years, respectively [12–14]. Although a previous study that externally validated the ARCTIC score included both medical and surgical patients, the median patient age was 58 years [15]. Conversely, our cohort consisted of mixed ICU patients with a median age of 71 years, and the frequency of ARC remained substantial even in patients aged > 65 years (21% [87/409]). Additionally, the large sample size of this study allowed the establishment of more detailed age thresholds. Age is a key risk factor for ARC, and these differences likely contribute to the higher sensitivity of the JPNARC score. Given that the therapeutic concentrations of drugs excreted by the kidneys are reduced in patients with ARC, sensitivity, rather than specificity, is more critical in predicting ARC in a critically ill population requiring high therapeutic drug levels. In this regard, the JPNARC score, while potentially overestimating the risk in a population with high predictive probability, offers the advantage of earlier detection of at-risk patients when compared with other scores.

The JPNARC score factors were more similar to the ARCTIC scores than the ARC scores (Table 3). The ARC score uniquely incorporated sepsis as a factor; however, even in our validation set, where sepsis was more frequent, its predictive performance was inadequate. Although several previous studies have reported sepsis as a risk factor for ARC, it was not selected as a critical factor in the meta-analysis [10]. Similarly, neither sepsis nor SOFA score contributed significantly to ARC development in our patient population. Severe sepsis is associated with a high incidence of renal dysfunction [5]. In this older mixed ICU population, sepsis was not significantly associated with the onset of ARC. In contrast, the ARCTIC score showed sufficient predictive ability in our cohort, indicating a robust association between ARC and younger age, male sex, and trauma, even in critically ill Japanese patients. However, the serum creatinine threshold for the JPNARC score was unexpectedly higher than anticipated. Racial differences in creatinine-based renal function estimation equations, particularly between Asian and non-Asian populations, have been reported [16, 17], primarily due to differences in muscle mass. We expected the serum creatinine threshold in the JPNARC score to be lower than that in the ARCTIC score, which was developed for a Western population; however, the opposite was found to be true. This discrepancy may also be attributed to the older baseline age of the study population. Further evaluation is needed to explore this hypothesis using baseline characteristics consistent with those of previous ARC studies.

The JPNARC score offers superior predictive performance and advantages in terms of labor and cost. Although ARC has been recognized as a phenomenon associated with drug treatment resistance in critical care settings, CrCl measurements in patients without renal impairment are rarely utilized. Nonetheless, creatinine- and cystatin C-based renal function estimation equations are not recommended because they tend to underestimate the measured CrCl levels in patients with ARC [18, 30, 31]. Similarly in the present study, creatinine-based estimated CrCl underestimated measured CrCl in patients with ARC and was not useful. Thus, using a simple JPNARC score would reduce the labor and costs associated with unnecessary CrCl measurements. As ARC is strongly linked to suboptimal pharmacokinetic outcomes [1–4], our model could facilitate the early detection of ARC and improve patient prognosis through timely intervention.

This study has several limitations. First, the results were not prospectively validated. Although we obtained a statistically adequate number of critically ill patients in the ARC prediction study, the high heterogeneity of the target population warrants a cautious interpretation of the findings. Second, as this was a single-center study, validation may have favored the JPNARC score. However, our study population was considerably larger than those used to develop existing scores, and external validation was performed. In addition, the validation set had a higher rate of sepsis, but the results were similar when these patients were excluded (data not shown). Further validation in larger populations is required to eliminate potential group bias. Third, the cohort study did not include TDM data on therapeutic drug monitoring. The goal of ARC studies is to prevent a reduction in therapeutic drug concentrations. However, it may be necessary to consider that many commonly used beta-lactams have not been monitored. Fourth, the study did not account for the time course of critical illness. The JPNARC score uses data at ICU admission to predict subsequent ARC onset; however, the time course of ARC varies across patients. Critically ill patients often experience notable muscle mass loss [32], which can reduce serum creatinine release over time. Therefore, caution is needed regarding the timing of the score evaluation. Accordingly, in-hospital emergency patients with prolonged histories of hospitalization were excluded from the analysis. Finally, treatment-related events were excluded from the analysis. Some patients in our study experienced a recurrence after an initial ARC pause, which could have affected the pharmacokinetics of subsequent renally excreted drugs. While drug doses are typically adjusted upward in patients with ARC, excessive drug exposure can worsen neurological outcomes [33]. Additionally, ARC duration varies widely among patients [18]. As it is difficult to predict the end of ARC, clinical decisions should be based on the patient’s day-to-day condition. Therefore, careful consideration of each patient’s treatment intensity and tolerability, particularly during the maintenance phase of drug therapy, is crucial.

## Conclusions

An integer-based scoring system was developed to predict the onset of ARC in a critically ill Japanese population and showed a high predictive performance. New models designed to predict the often-unrecognized ARC phenomenon may aid in the decision-making process for upward drug dosage modifications, especially in resource- and labor-limited settings.

## Data Availability

The datasets used and/or analyzed in the current study are available from the corresponding author upon reasonable request.

## Abbreviations

ARC: Augmented renal clearance
CrCl: Creatinine clearance
95% CI: 95% Confidence interval
ICU: Intensive care unit
ARCTIC: Augmented Renal Clearance in Trauma Intensive Care
CNS: Central nervous system
AUROC: Area under the receiver operating characteristic curve
PPV: Positive predictive value
NPV: Negative predictive value
